# Ethylene signaling involves in seeds germination upon submergence and antioxidant response elicited confers submergence tolerance to rice seedlings

**DOI:** 10.1186/s12284-019-0284-z

**Published:** 2019-04-11

**Authors:** Yi-Chun Huang, Tsun-Hao Yeh, Chin-Ying Yang

**Affiliations:** Department of Agronomy, National Chung Hsing University, Taichung, 40227 Taiwan

**Keywords:** Rice, Submergence, Ethylene, Reactive oxygen species, Antioxidant enzyme activity

## Abstract

**Background:**

Flooding has negative impact on agriculture. The plant hormone ethylene is involved in plant growth and stress responses, which are important role in tolerance and adaptation regulatory mechanisms during submergence stress. Ethylene signaling crosstalk with gibberellin signaling enhances tolerance in lowland rice (Flood Resistant 13A) through a quiescence strategy or in deepwater rice through an escape strategy when rice is submerged. Information regarding ethylene-mediated priming in submergence stress tolerance in rice is scant. Here, we used 1-aminocyclopropane-1-carboxylic acid, an ethylene precursor, to evaluate the response in submerged rice seedlings.

**Results:**

The germination rate and mean germination times of rice seeds was higher in seedlings under submergence only when ethylene signaling was inhibited by supplemented with silver nitrate (AgNO_3_). Reduced leaf chlorophyll contents and induced senescence-associated genes in rice seedlings under submergence were relieved by pretreatment with an ethylene precursor. The ethylene-mediated priming by pretreatment with an ethylene precursor enhanced the survival rate and hydrogen peroxide (H_2_O_2_) and superoxide (O_2_^−^) anion accumulation and affected antioxidant response in rice seedlings.

**Conclusions:**

Pretreatment with an ethylene precursor leads to reactive oxygen species generation, which in turn triggered the antioxidant response system, thus improving the tolerance of rice seedlings to complete submergence stress. Thus, H_2_O_2_ signaling may contribute to ethylene-mediated priming to submergence stress tolerance in rice seedlings.

**Electronic supplementary material:**

The online version of this article (10.1186/s12284-019-0284-z) contains supplementary material, which is available to authorized users.

## Background

Severe climate-related disasters include flooding due to increased frequency of heavy rain. Flooding affects agriculture causing outright crop yield losses. The term flooding comprises both waterlogging and submergence. The pore space in the soil is filled with water when soil is under excess water stress; this decreases soil oxygen levels, limits gas diffusion and soil nutrient effusion, and impairs plant growth and development (Nishiuchi et al. 2012). With increased submergence duration, tiller number, green leaves number, and dry weight of rice decreases. The survival and growth of rice are severely affected by submergence (Reddy and Mittra 1985; Gautam et al. 2017; Wu and Yang 2016).

Unfavorable conditions activate phytohormonal signals in plants, in turn enhancing their tolerance to environmental stress. The gaseous plant hormone ethylene mediated developmental processes and stress tolerance, such as seed germination, senescence, and stress responses (Yu et al. 2017; Xia et al. 2015; El-Maarouf-Bouteau et al. 2015). Ethylene signaling-induced mitogen-activated protein kinase (MAPK) cascades can be activated with the application of the ethylene precursor 1-aminocyclopropane-1-carboxylic acid (ACC) in *Medicago* and *Arabidopsis* (Ouaked et al. 2003). Under salt stress, ethylene can activate the MAPK cascade and enhance reactive oxygen species (ROS) generation (Teige et al. 2004). The interplay of ethylene signaling and ROS production activates the antioxidant defense system for flooding responses in rice (Steffens 2014; Yang and Hong 2015).

Several studies have reported that ethylene is crucial against hypoxia signal-inducing flooding stress. Aerenchyma formation can be induced in maize roots by applying ethylene in flooded conditions (Rajhi et al. 2011). Ethylene signaling triggers the process of programmed cell death resulting in ethylene-responsive lysigenous aerenchyma formation (Guo et al. 2015; Muhlenbock et al. 2007; Chen et al. 2002). Two key ethylene biosynthesis enzymes, 1-aminocyclopropane-1-carboxylic acid synthase (ACS) and 1-aminocyclopropane-1-carboxylic acid oxidase (ACO), are involved in plant response to hypoxia stress. ACS converts S-adenosylmethionine (AdoMet) into ACC and the byproduct 5′-methylthioadenosine; then, ACO converts ACC to ethylene, thus increasing the ethylene levels (Rzewuski and Sauter 2008; Adams and Yang 1979). In this study, to further clarify the role of ethylene signaling during submergence, we used ACC, an ethylene precursor, to evaluate the response in submerged rice seedlings.

## Results

### Submergence-induced germination inhibition was alleviated after ethylene signaling was blocked

**Fig. 1:**
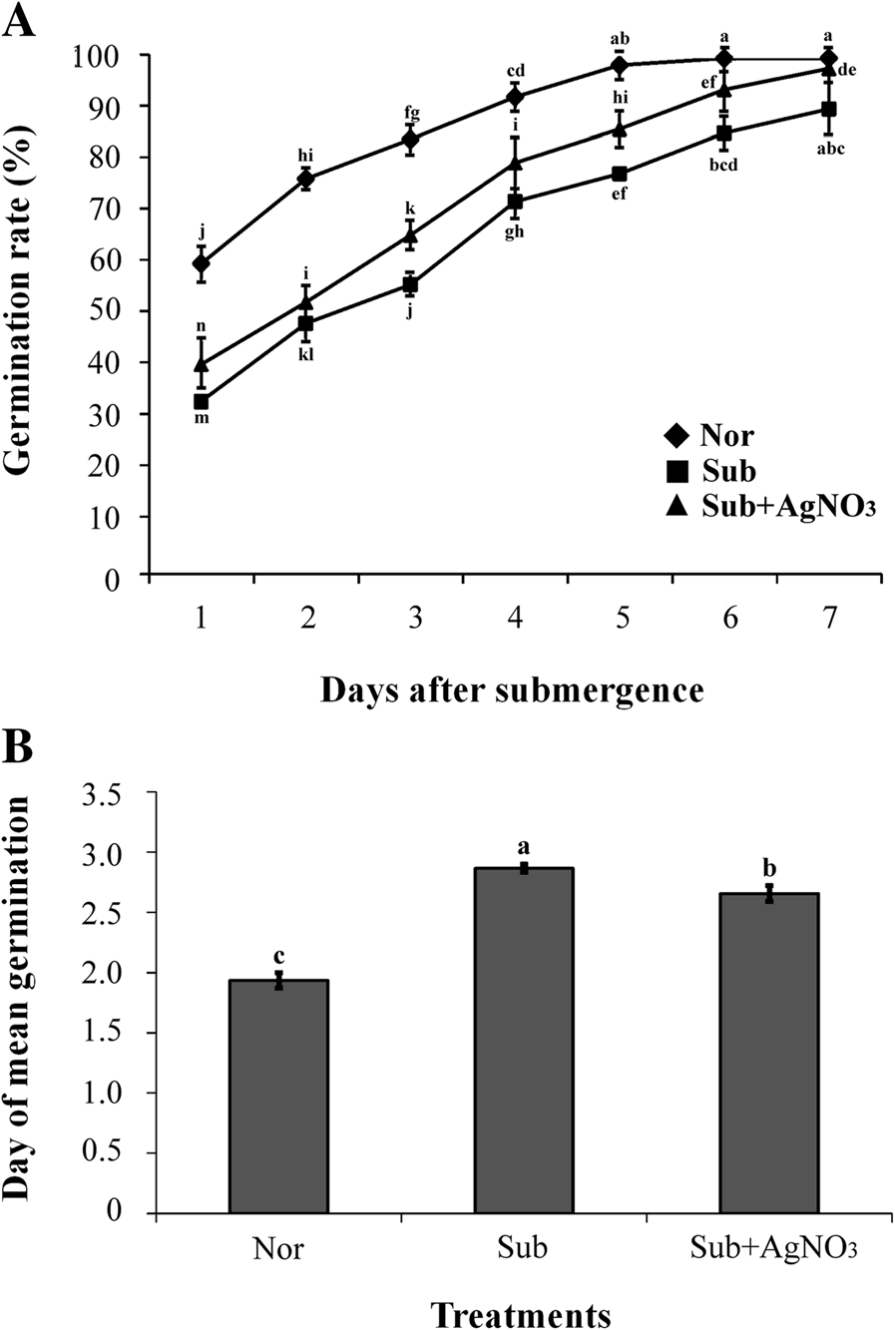
The germination assay of rice seeds under submergence combined with inhibition of ethylene signaling conditions. Seeds were surface sterilized and placed on a wet filter paper under Nor, Sub, and Sub + AgNO_3_ for pretreatment for 2 days and subsequently placed under Nor in a growth chamber for the germination assay. Seed germination was observed for 1, 2, 3, 4, 5, 6, and 7 days. **a** The germination rate of TK9 rice seeds. **b** The day of mean germination. The data represent the average values ± SD from 30 seedlings of each treatment obtained from three biologically independent experiments. The values with different letters are significantly different at *P* < 0.05, according to one-way ANOVA with post hoc Duncan’s test

To evaluate the effect of ethylene signaling on rice seed germination during submergence, we pretreated rice seeds under normoxia (Nor), submergence (Sub), and submergence supplemented with silver nitrate (Sub + AgNO_3_) for 2 days, and then, we again placed the seeds under normal condition to calculate the seed germination rates and mean germination times (MGTs). Ag^+ 2^ ions in AgNO_3_ inhibit the action of ethylene by reducing the ethylene receptor’s capacity to bind ethylene (Yang 1985; Kumar et al. 2009). Our results presented that the seeds pretreated under submergence germinated slower than those under Nor. However, germination inhibition was alleviated under submergence only when supplemented with AgNO_3_ (Fig. 1a). MGT of seeds pretreated under Nor, Sub, and Sub + AgNO_3_ was 1.94, 2.87, and 2.66 days, respectively (Fig. 1b). Thus, submergence combined with the ethylene signaling inhibition reduced MGT.

### Survival rate was enhanced after pretreatment with ethylene precursor in rice seedlings subjected to submergence stress

**Fig. 2:**
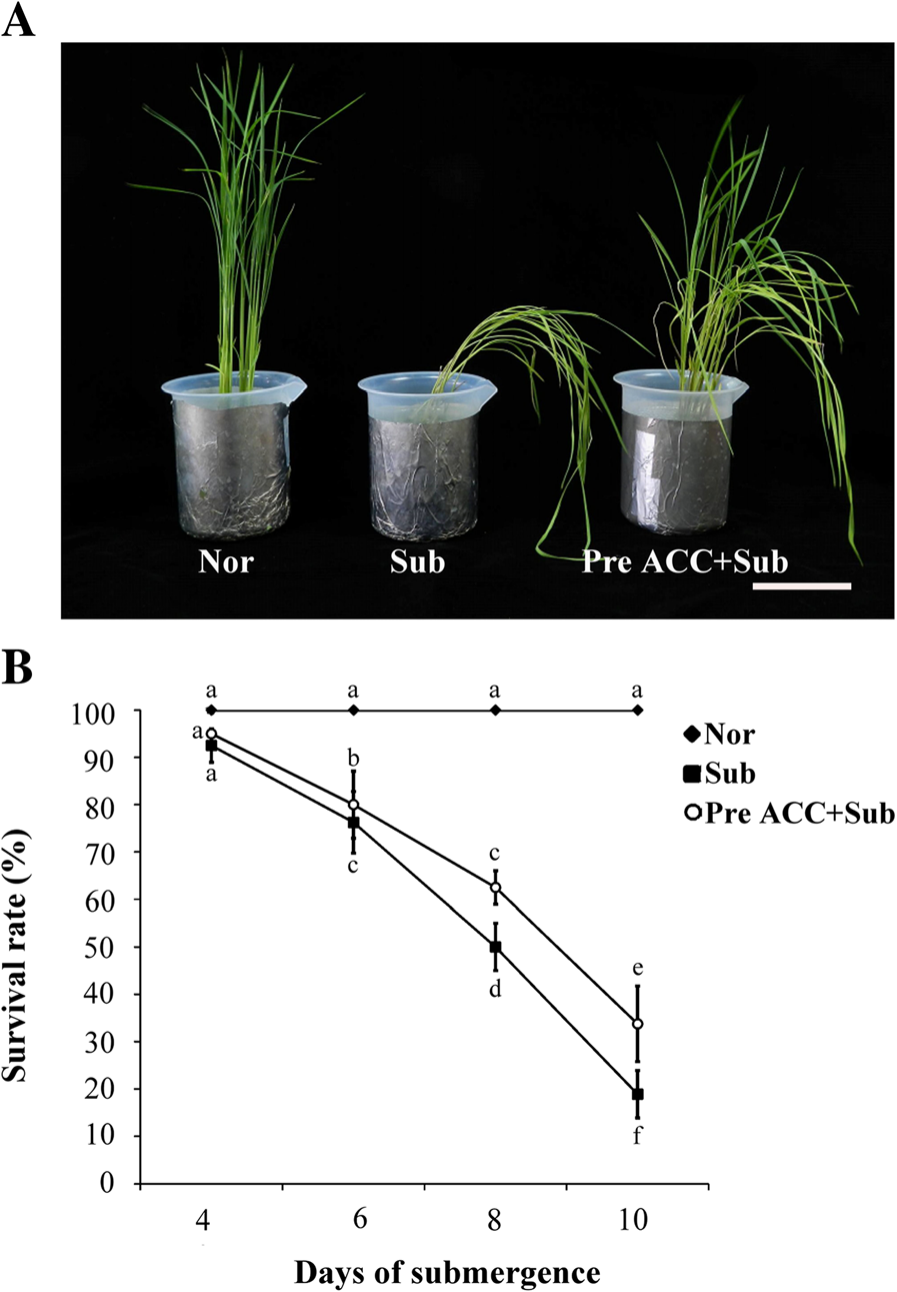
Tolerance determination of rice seedlings under submergence by pretreatment with an ethylene precursor. **a** The phenotypes of 8-day-old TK9 rice seedlings treated under Nor, Sub, and Pre ACC + Sub for 2 days and then subjected to submergence for 10 days and subsequent recovery for 10 days. Bar = 1 cm. The photograph exhibits the results in three independent seedlings. **b** The survival rate of TK9 rice seedlings treated under Nor, Sub, and Pre ACC + Sub for 2 days and then subjected to submergence for 4, 6, 8, and 10 days followed by 10-day recovery. The data represent the average values ± SD from 30 seedlings of each treatment obtained from three biologically independent experiments. The values with different letters are significantly different at *P* < 0.05, according to one-way ANOVA with post hoc Duncan’s test

Ethylene, involved in hypoxia signaling, affects anaerobic gene expression and ethanolic fermentation in plants (Yang et al. 2011; Peng et al. 2001). To investigate the effects of ethylene-mediated priming on submergence stress tolerance, the 8-day-old Tai-keng 9 (TK9) rice seedlings were grown on Kimura B medium with or without ACC pretreatment for 2 days; then, they were subjected to complete submergence for 4, 6, 8, and 10 days and finally allowed to recover for 10 days. The survival rate was assessed on the basis of ability to form one or more new leaves. ACC is an effective precursor of ethylene in higher plants. The survival rates of TK9 rice seedlings without and with ACC pretreatment followed by complete submergence for 4, 6, 8, and 10 days were 92.5%, 76.3%, 50.0%, and 18.8%, respectively, and 95.0%, 80.0%, 62.5%, and 37.8%, respectively (Fig. 2a and b). Thus, survival rates significantly increased after pretreatment with an ethylene precursor in TK9 seedlings under submergence stress.

### Reduced leaf chlorophyll contents and induced senescence-associated genes in rice seedlings under submergence were alleviated after ethylene precursor pretreatment

**Fig. 3:**
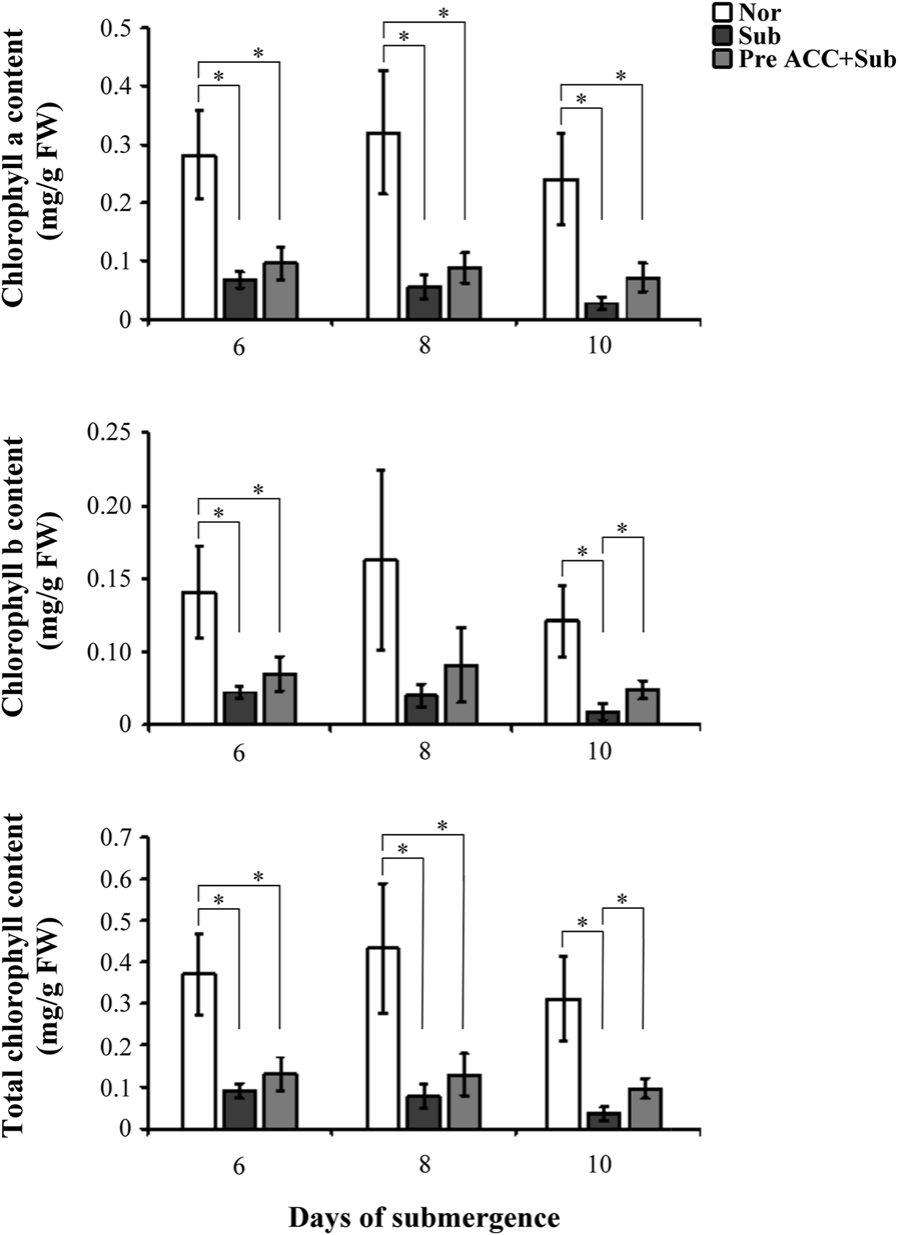
The chlorophyll content of rice seedlings by pretreatment with an ethylene precursor under submergence conditions. The chlorophyll a, b and total content of 8-day-old TK9 rice seedlings treated under Nor, Sub, and Pre ACC + Sub for 2 days and then subjected to submergence for 4, 6, 8, and 10 days. The data represent the average values ± SD from four biologically independent experiments. The values with asterisk are significantly different at *P* < 0.05, according to Student’s *t* test

The chlorophyll contents and senescence-assocaited genes (SAGs) are severely affected under complete submergence (Wu and Yang 2016). To understand the effects of ethylene-mediated priming in chlorophyll contents during submergence, the 8-day-old TK9 rice seedlings were pretreated with or without ACC for 2 days and then subjected to complete submergence for 6, 8, and 10 days to measure chlorophyll contents. Seedlings pretreated ACC with under complete submergence demonstrated higher chlorophyll b and total chlorophyll content than did untreated seedlings under submergence (Fig. 3).

**Fig. 4:**
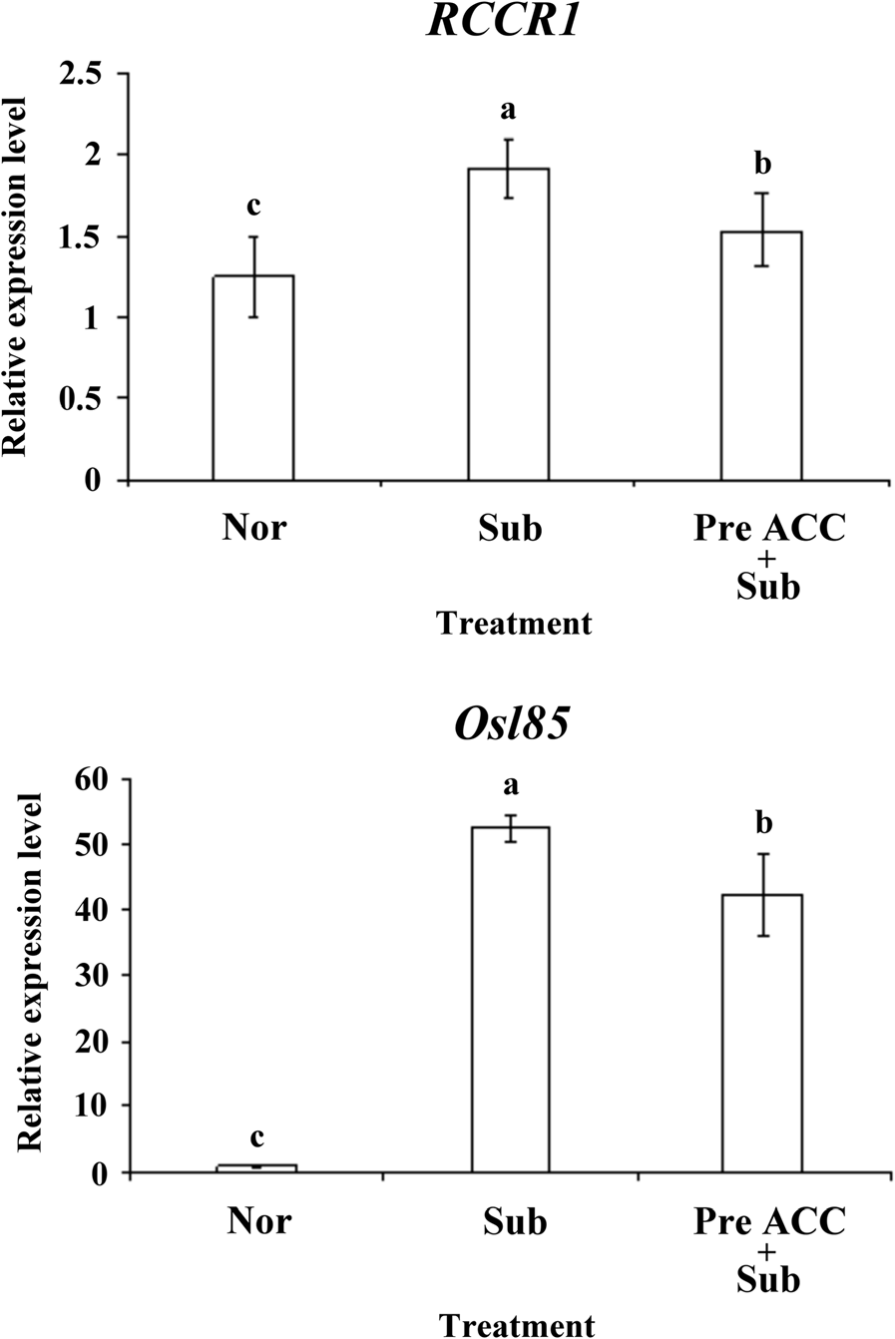
The transcript levels of SAGs in rice seedlings by pretreatment with an ethylene precursor under submergence conditions. qRT-PCR quantified the transcript levels of *RCCR1* and *Osl85* genes in 8-day-old TK9 rice seedlings treated under Nor, Sub, and Pre ACC + Sub for 2 days and then subjected to submergence for 4 days. The data represent the average values ± SD from three biologically independent experiments. The values with different letters are significantly different at *P* < 0.05, according to one-way ANOVA with post hoc Duncan’s test

The mRNA expression of the SAGs red chlorophyll catabolite reductase 1 (*RCCR1*; involved in chlorophyll degradation) (Pruzinska et al. 2007), and isocitrate lyase (*Osl85*; highly induced by prolonged darkness and natural senescence) (Yamada et al. 2014) was determine through quantitative reverse transcription polymerase chain reaction (qRT-PCR). The results indicated that the induction of *RCCR1* and *Osl85* expression was significantly lower in seedlings under complete submergence with ACC pretreatment than in those under submergence only (Fig. 4). We also had detected some genes expression that involved in chlorophyll biosynthesis and degradation such as *CAO1*, *HEMA1*, *NYC1* and *NOL* by qRT-PCR. The results presented no significant different in our experiment treatment (Additional file 1: Figure S1). Thus, pretreatment of rice seedlings with an ethylene precursor significantly affected the reduction of leaf chlorophyll content and induction of SAG expression under submergence stress.

### Pretreatment with an ethylene precursor lead to hydrogen peroxide and superoxide ion accumulation and affected antioxidant response during submergence

**Fig. 5:**
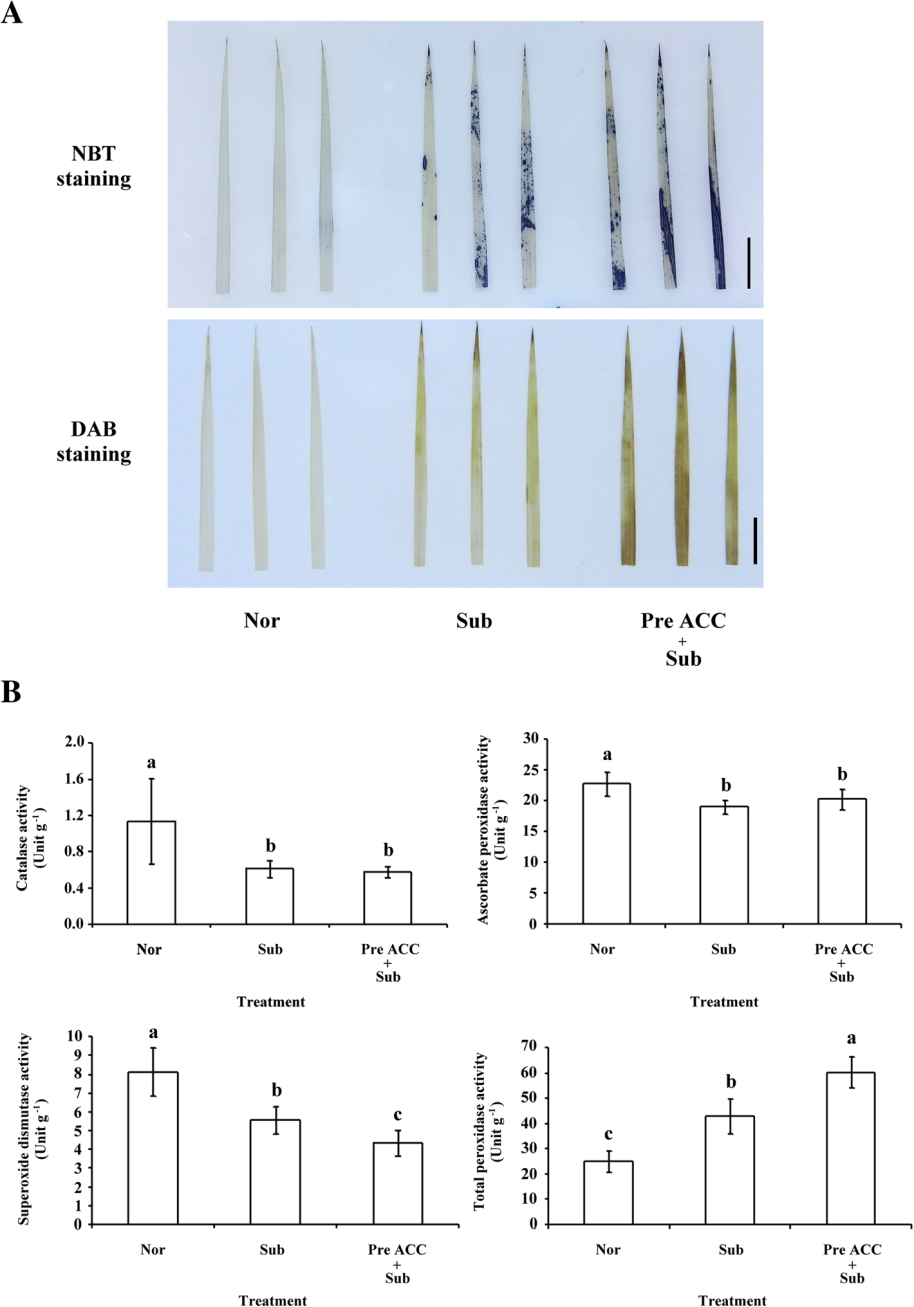
The detection of O_2_^−^ and H_2_O_2_ accumulation and antioxidative enzyme activity assay in rice seedlings pretreated with an ethylene precursor under submergence conditions. **a** The NBT and DAB staining methods for detecting the accumulation of O_2_^−^ and H_2_O_2_ in detached leaves of 8-day-old TK9 rice seedlings treated under Nor, Sub, and Pre ACC + Sub for 2 days and then subjected to submergence for 4 days. Same results were obtained in three independent experiments. Photograph exhibits results of three independent leaves. Bar = 1 cm. **b** The enzyme activity of detached leaves of 8-day-old TK9 rice seedlings treated under Nor, Sub, and Pre ACC + Sub for 2 days and then subjected to submergence for 4 days. CAT, APX, SOD, and POX activities are the average values ± standard deviation of three biologically independent experiments each with duplicate samples. The values with different letters are significantly different at *P* < 0.05, according to a one-way ANOVA with post hoc Duncan’s test

ROS is involved in ethylene-dependent and -independent submergence adaptation (Yang and Hong 2015). To determine how ethylene-mediated priming affects antioxidant system homeostasis, ROS accumulation and antioxidant enzyme activity were detected. The 8-day-old TK9 rice seedlings were pretreated with and without ACC for 2 days and then subjected to complete submergence for 4 days; superoxide ion (O_2_^−^) and hydrogen peroxide (H_2_O_2_) accumulation was detected by staining with nitro blue tetrazolium (NBT) and 3,3′-diaminobenzidine (DAB). The staining of first leaf revealed higher O_2_^−^ and H_2_O_2_ accumulation under complete submergence after pretreatment with ACC than under submergence alone (Fig. 5a).

The activities of antioxidative enzymes, namely catalase (CAT), ascorbate peroxidase (APX), superoxide dismutase (SOD), and total peroxidase (POX), were then determined after complete submergence with or without ACC pretreatment. The CAT, SOD, and APX activities decreased under submergence stress with or without ACC pretreatment. However, SOD activity significantly decreased after ACC pretreatment under submergence. POX activity increased under submergence stress; it was particularly higher under submergence after ACC pretreatment (Fig. 5b). Thus, pretreatment with an ethylene precursor may affect intracellular redox homeostasis and antioxidant systems under submergence stress.

## Discussion

The plant hormone ethylene plays important roles in plant adaptation to submergence stress. It is the principal factor initiating fast underwater elongation of leaves or stems—the so-called escape strategy in deepwater rice (Hattori et al. 2011). In lowland rice Flood Resistant 13A, it is elicits a quiescence strategy based on suppression of elongation to avoid energy consumption during flash flooding (Manzur et al. 2009). Studies presented that some QTLs (quantitative trait loci) associated with tolerance of flooding during germination have been identified that revealed ABA and GA involved in submergence tolerance during germination (Miro and Ismail, 2013). The role of ethylene in priming during submergence stress remains ambiguous. To further understand the effects of ethylene-mediated priming on tolerance and antioxidant response to submergence stress, we used an ethylene precursor ACC to investigate change in physiological and molecular responses in rice seedlings under submergence stress. Studies have revealed the roles of ethylene in the release of primary and secondary dormancy and the germination of nondormant seeds under normal and stressed conditions in many plant species (Petruzzelli et al. 2000; Kepczynski and Kepczynska 1997); poor germination is a feature of the *Arabidopsis* ethylene-insensitive mutant (Johnson and Ecker 1998). Waterlogging or submergence causes a rapid decline in dissolved oxygen concentrations in the soil water, thus resulting in seed germination failure and lengthening the germination time in pea, oak, and lupin seeds (Sarlistyaningsih et al. 1995; Perez-Ramos and Maranon 2009; Jackson and Hall 1987). In the current study, the germination rate of TK9 rice seeds considerably decreased but their MGT considerably increased under the Sub condition compare with that under the Nor condition (Fig.1 a and b). However, germination inhibition was partially disrupted under submergence in Sub + AgNO_3_-treated seedlings because ethylene signaling was inhibited through blocking ethylene perception with silver ions (Fig. 1 a and b). Therefore, the results imply that in addition to ethylene signaling, other pathways are involved in the regulation of seed germination under submergence stress. Studies indicated that silver ions affects not only ethylene signaling may also auxin efflux to affect root elongation (Strander et al., 2009). Whether the auxin signaling involved in the regulation of seed germination under submergence stress need further research.

Complete submergence of rice plants can severely delay physiological responses, retard growth and development, reduce yield, and even cause death (Jackson and Ram 2003; Yang et al. 2017). We demonstrated that the survival rate of rice seedlings can be improved under complete submergence stress through ACC pretreatment (Fig. 2). Moreover, under submergence, chlorophylls b and total chlorophyll contents could be maintained by ACC pretreatment (Fig. 3). Under complete submergence, ACC-pretreated seedlings demonstrated lower SAG mRNA expression than did untreated seedlings (Fig. 4). Thus, ethylene-mediated priming has senescence inhibition-associated positive effects on plant submergence tolerance.

ROS plays a key role in signal transduction in cells (Mittler et al. 2011). Homeostatic regulation of ROS–antioxidant interactions in plant cells confers increased to environmental stress tolerance to the plants. Cellular antioxidants influence plant growth and development by modulating processes from cell division and cell elongation to senescence and death (Foyer and Noctor 2005). In plants, complex intracellular mechanisms regulate the ROS production and scavenging, particularly under stress. In our study, H_2_O_2_ accumulation and POX activity increased under submergence with ACC pretreatment compared with that under submergence alone (Fig. 5). These results may imply that H_2_O_2_ signaling contributes to ethylene-mediated priming on submergence tolerance in rice seedlings. Taken together, this study demonstrated that ACC pretreatment trigger positive priming mechanism to increase plant tolerance to submergence.

## Conclusions

In conclusion, our results demonstrated that the germination rates of rice seeds under submergence partially increased after ethylene signaling was inhibited. In rice seedlings, ethylene-mediated priming through pretreatment with an ethylene precursor modulated leaf chlorophyll content and SAG expression, enhanced survival, increased H_2_O_2_ and O_2_^−^ accumulation, and reduced antioxidant response was affected by. Thus, seed germination and rice seedling tolerance can be improved under complete submergence by modulating ethylene signaling because ethylene-mediated priming affects senescence induction and ROS and antioxidant response conferring submergence tolerance to rice. Whether this regulatory mechanism can crosstalk with other pathways remains unclear and merits further study.

## Materials and methods

### Plant materials and growth conditions

TK9 rice (*Oryza sativa* Japonica) was used in this study. Rice seeds were sterilized by dipping in 3% sodium hypochlorite solution for 30 min, followed by gentle washing with distilled water for at least four to five times. The sterilized seeds were subsequently placed on a wet filter paper for 3 days at 28 °C under a 16-h light–8-h dark cycle in a growth chamber. The germinated seeds were transferred onto a metal grid placed over a 500-mL beaker containing Kimura B medium for growth. For the seed germination assay, data were collected from 30 seeds for each treatment in three independent experiments. Seeds were placed under Nor, in 4.5-cm-deep water in a water tank (Sub), or in 4.5-cm-deep water containing 10 μM AgNO_3_ in a water tank (Sub + AgNO_3_) for pretreatment for 2 days; then, the seeds were transferred onto a wet filter paper for germination. Germination was confirmed when the radicles were 1 mm long. Germination percentage was recorded every 24 h for 7 days. The number of germinated seeds was expressed as a percentage of the total number of seeds plated for the indicated periods. MGTs were calculated to assess the time required for germination (Matthews and Khajeh-Hosseini 2007).

### Seedlings submergence, ACC treatment, and survival rate determination

For submergence treatment, 8-day-old seedlings were completely submerged with or without 10 μM ACC pretreatment for 2 days, followed by transferring into a water tank (40 × 40 × 60 cm^3^) with 55-cm-deep water for 4, 6, 8, and 10 days under a 16-h light–8-h dark cycle. Under Nor, seedlings were placed under normal condition for the indicated periods. The water was drained out for subsequent 10-day recovery. The ability to grow new leaves after 10-day recovery was considered the measure of survival. Experiments were repeated three times, and at least 30 seedlings were measured independently each time. After each treatment, samples tissues were immediately frozen in liquid nitrogen and stored at − 80 °C for further assay.

### Plant chlorophyll content measurements and qRT-PCR analyses

For chlorophyll content assay, the 8-day-old seedlings were treated under Nor and Sub with and without 10 μM ACC pretreatment for 2 days followed by submergence for 6, 8, and 10 days. Above-ground tissue (50 mg) was collected and ground in 2 mL of sodium phosphate buffer (50 mM, pH 6.8); 40 μL of this solution was added to 960 mL of 99% ethanol and incubated for 30 min at room temperature in the dark with gentle shaking. After centrifugation at 4 °C for 15 min at 1000 g, the absorbance of the supernatant was measured at 665 and 649 nm using a spectrophotometer (Metertec SP8001) for determining chlorophyll a and b and total chlorophyll contents. The values were collected from three biologically independent experiments.

For qRT-PCR analyses, total RNA was extracted using TRIzol (Invitrogen, Carlsbad, CA, USA) and then subjected to DNase treatment using the TURBO DNA-free Kit (Ambion, Austin, TX, USA). RNA concentration was determined, and samples were then reverse transcribed into cDNA by using Moloney murine leukemia virus reverse transcriptase (Invitrogen). qRT-PCR was performed as previously described (Yang et al. 2017) using a Bio-Rad CFX instrument (CFX Connect™, Bio-Rad, USA) with Power SYBR Green PCR Master Mix (GeneMark, Taipei, Taiwan), according to the manufacturer’s recommendations. The ubiquitin gene was used as an internal control for normalization. Relative expression levels were analyzed using Bio-Rad CFX Manager (version 3.1). Experiments were repeated three times independently with duplicate samples. The primer sequences for qRT-PCR are presented in Table 1.

**Table 1:** Primers used for quantitative RT-PCR experiments

Gene name	Primer sequence
*Osl85* - forward	5′-catgggcaaaggagttactgaagag − 3′
*Osl85* - reverse	5′-ggatttggcaagaacatggctg − 3’
*OsRCCR1* - forward	5′-gcaccttctcactgacagcaatac − 3’
*OsRCCR1* - reverse	5′-accacgcactatctcttccaagg − 3’
*Osubiquitin* - forward	5′-aaccagctgaggcccaaga-3’
*Osubiquitin* - reverse	5′-acgattgatttaaccagtccatga-3’
*OsCAO1* - forward	5′-tgctcatcaagccttccttcaggtg-3’
*OsCAO1* - reverse	5′-ctcgactgatacgtttcttgttgcg-3’
*OsHEMA1* - forward	5′-aggaaagaagtagcatagcg-3’
*OsHEMA1* - reverse	5′-cgatagagtcttgagtggtc-3’
*OsNYC1* - forward	5′-cacactgcttctcctggaatgg-3’
*OsNYC1* - reverse	5′-ctacgctcaacactttccttcacc-3’
*OsNOL* - forward	5′-ccacgaaaggtataggatatg-3’
*OsNOL* - reverse	5′-tcaagtcagtcaccgcagat-3’

### Histochemical staining and antioxidative enzyme activity assay

The detached leaves of 8-day-old rice seedlings were treated under Nor, Sub, and submergence supplemented with 10 μM ACC (Pre ACC + Sub) for 2 days and then submerged for 4 days. O_2_^−^ and H_2_O_2_ accumulation in cells was observed through the NBT and DAB staining methods, as previously described (Yang and Hong 2015). The results were obtained from three independent experiments. For the antioxidative enzyme assay, shoot tissue (50 mg) was excised and immediately used for enzyme extraction. The levels of CAT, APX, POX, and SOD activity were analyzed as previously described (Wu and Yang 2016). Each experiment was repeated three times.

## Additional file

Additional file 1: **Figure S1.** The transcript levels of related chlorophyll metabolism in rice seedlings by pretreatment with an ethylene precursor under submergence conditions were detected by qRT-PCR (JPG 1103 kb)

## Abbreviations

ACC: 1-Aminocyclopropane-1-carboxylic acid
APX: Ascorbate peroxidase
CAT: Catalase
H_2_O_2_: Hydrogen peroxide
POX: Total peroxidase
ROS: Reactive oxygen species
RT-PCR: Reverse transcriptase-polymerase chain reaction
SAGs: Senescence-associated genes
SOD: Superoxide dismutase
