# Supplementary figures and images for: Ethylene signaling involves in seeds germination upon submergence and antioxidant response elicited confers submergence tolerance to rice seedlings
Source: Rice (N Y). 2019 Apr 11;12:23. doi: 10.1186/s12284-019-0284-z (PMC6458221; doi:10.1186/s12284-019-0284-z)

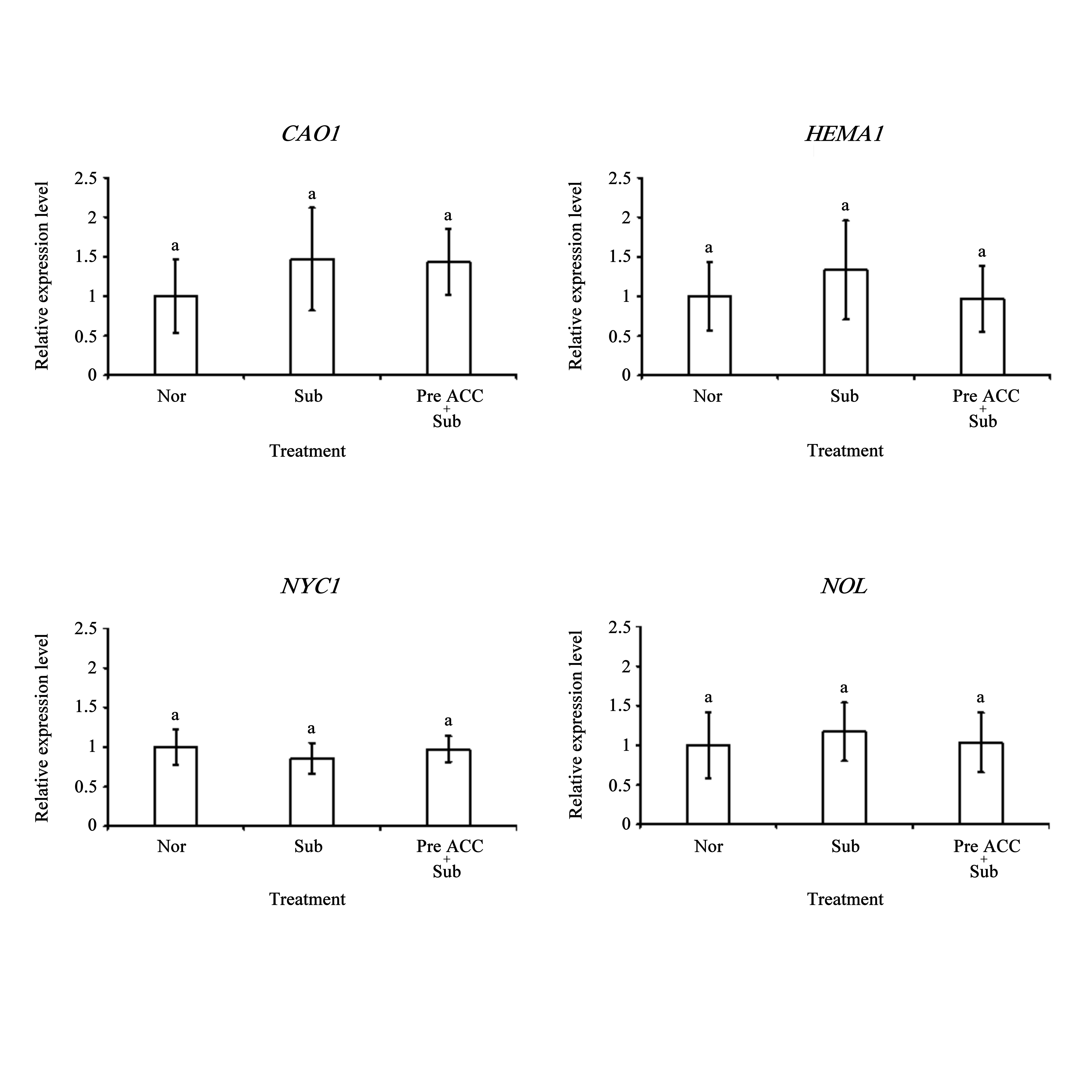

Supplement: Supplementary file 1 — Figure S1. The transcript levels of related chlorophyll metabolism in rice seedlings by pretreatment with an ethylene precursor under submergence conditions were detected by qRT-PCR (JPG 1103 kb) [file 12284_2019_284_MOESM1_ESM.jpg]
